# Transcriptional analysis of the *bglP *gene from *Streptococcus mutans*

**DOI:** 10.1186/1471-2180-6-37

**Published:** 2006-04-21

**Authors:** Christopher K Cote, Allen L Honeyman

**Affiliations:** 1 Department of Medical Microbiology and Immunology, University of South Florida, College of Medicine, Tampa, Florida 33612, USA; 2 Bacteriology Division, USAMRIID, 1425 Porter Street, Fort Detrick, Frederick, MD 21702, USA; 3 Department of Biomedical Sciences, Texas A&M University System Health Science Center, Baylor College of Dentistry, 3302 Gaston Avenue, Dallas, Texas 75246, USA

## Abstract

**Background:**

An open reading frame encoding a putative antiterminator protein, LicT, was identified in the genomic sequence of *Streptococcus mutans*. A potential ribonucleic antitermination (RAT) site to which the LicT protein would potentially bind has been identified immediately adjacent to this open reading frame. The *licT *gene and RAT site are both located 5' to a beta-glucoside PTS regulon previously described in *S. mutans *that is responsible for esculin utilization in the presence of glucose. It was hypothesized that antitermination is the regulatory mechanism that is responsible for the control of the *bglP *gene expression, which encodes an esculin-specific PTS enzyme II.

**Results:**

To localize the promoter activity associated with the *bglP *locus, a series of transcriptional *lacZ *gene fusions was formed on a reporter shuttle vector using various DNA fragments from the *bglP *promoter region. Subsequent beta-galactosidase assays in *S. mutans *localized the *bglP *promoter region and identified putative -35 and -10 promoter elements. Primer extension analysis identified the *bglP *transcriptional start site. In addition, a terminated *bglP *transcript formed by transcriptional termination was identified via transcript mapping experiments.

**Conclusion:**

The physical location of these genetic elements, the RAT site and the promoter regions, and the identification of a short terminated mRNA support the hypothesis that antitermination regulates the *bglP *transcript.

## Background

Beta-glucosides are carbohydrates that contain a core glucose molecule that has an R-group attached to the C-l carbon. The R-group may be another carbohydrate or various other molecules such as amino acids or ringed compounds. Beta-glucosides are commonly found in nature in various sources such as tuberous vegetables. These compounds are generally broken down for the glucose molecule, while the R-group may or may not be metabolized. Beta-glucoside utilization systems have been described in several bacteria including *Escherichia coli *[1], *Erwinia chrysanthemi *[2], *Clostridium longisporum *[3], *Lactobacillus plantarium *[4], *Bacillus subtilis *[5,6], and *Streptococcus mutans *[7]. All of these organisms rely on the phosphoenolpyruvate-dependent phosphotransferase system (PTS) [8] for the transport of beta-glucosides into the bacterial cell.

It has been previously shown that the *S. mutans bglP *gene encodes a beta-glucoside-specific enzyme II of the PTS [7]. An additional open reading frame adjacent to *bglP *has been isolated, sequenced, and examined for a possible regulatory function [9]. The open reading frame encodes a putative regulatory protein of the BglG family of transcriptional antiterminators [10]. Antiterminator proteins are believed to be involved in the transcriptional regulation of the beta-glucoside-specific genes present within *E. coli *[1], *E. chrysanthemi *[2], *L. lactis *[11], *L. plantarium *[4], *B. subtilis *[5,6], and *C. longisporum *[3]. This type of antiterminator protein has been shown to bind to a ribonucleic antitermination (RAT) site [12,13], which is a palindromic sequence that may form multiple hairpin structures in the mRNA, some of which would act as a transcriptional terminator. Binding of one of these potential RNA structures by the antiterminator protein prevents the formation of the transcription terminator hairpin structure [14] and results in the elongation of the mRNA via transcriptional read through into the region encoding the beta-glucoside-specific gene product that is not normally transcribed. Thus, the mechanism of transcriptional regulation by antitermination allows for the expression of the beta-glucoside-specific loci only in the presence of a beta-glucoside [14].

The molecular mechanisms behind the process of antitermination have been studied most extensively in *E. coli*. and *B. subtilis*. The *bglGFB *operon in *E. coli *encodes an antiterminator protein (*bglG*), an EII component of the PTS that is specific for beta-glucosides (*bglF*), and a phospho-beta-glucosidase (*bglB*) [1]. The BglG protein is responsible for binding two transcription terminators flanking the *bglG *gene [1,15], which results in the production of a full-length transcript and full induction of the *bgl *operon [16]. It has subsequently been shown that the EII protein (BglF) also plays a regulatory role in antitermination [17].

The molecular mechanisms responsible for the regulation of beta-glucoside-specific loci in *B. subtilis *by the LicR and LicT proteins are less documented. The *licBCAH *operon in *B. subtilis *has been shown to be responsible for the catabolism of cellobiose and lichenan [6]. The *licBCA *genes encode the EII component of the PTS and a phospho-beta-glucosidase is encoded by the *licH *gene [6]. This *lic *operon was determined to be regulated in part by the LicR protein [6]. The LicR protein has been shown to contain several PTS regulation domains (PRDs) [10], and, presumably, the LicR protein is stimulated upon the phosphorylation of these PRDs [18]. The activity of the LicR protein has also been shown to be negatively effected by phosphorylation of the EIIA domain encoded by the *licBCA *genes [18].

*B. subtilis *has a second beta-glucoside specific locus, the *bglPH *operon, that is responsible for the breakdown of the beta-glucosides salicin and arbutin [5]. The *bglPH *operon, encoding the EII component of the PTS (*bglP*) and a phospho-beta-glucosidase (*bglH*), is regulated by the LicT antiterminator protein [19]. The LicT protein has been shown to be phosphorylated by HPr-phosphate [20], which allows the formation of an active LicT dimer [21].

**Figure 1:**
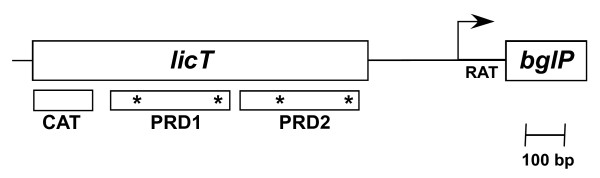
Diagram of the *licT *gene structure. The locations of the conserved co-antiterminator (CAT) domain and PTS regulatory domains (PRDs) are indicated by the boxed regions below the *licT *coding sequence. The asterisks indicate the positions of conserved histidine residues that are potentially phosphorylated to regulate the activity of the LicT protein. The start site of the *bglP *transcript is shown by the arrow. The RAT site is located between the start site of transcription and the coding region of the *bglP *gene.

We have previously determined that the *S. mutans *LicT protein functions as regulator of various genes within the *bglP *regulon [9]. The gene encoding the LicT protein is located immediately 5' to a putative RAT binding site and the *bglP *gene (Figure 1). The LicT protein, which is highly homologous to other antiterminator proteins, has a co-antiterminator domain (CAT) [12] and two PTS regulatory domains (PRDs) [10] (Figure 1). CAT domains form symmetrical dimers that bind RNA and are only found in antitermination proteins that regulate sugar uptake and metabolism in bacteria [12]. The LicT PRD domains contain potential sites for phosphorylation by the molecules that would putatively regulate LicT activity, either HPr-P or an Enzyme II-P [10]. Thus, the *S. mutans bglP *gene is potentially regulated by antitermination by the LicT protein.

**Figure 2:**
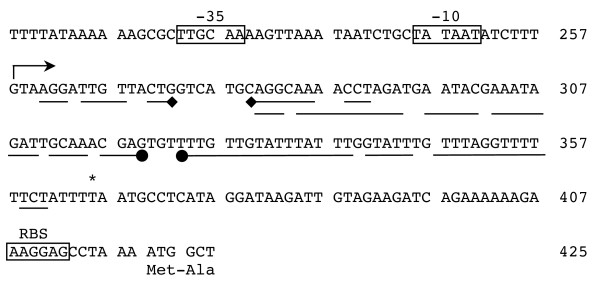
Diagram of the *bglP *promoter region nucleotide sequence. The DNA sequence of bases 208 to 425 of the *XbaI*-*Hind*III restriction fragment displaying regulation promoter activity is shown. Boxes indicate the position of the putative -35, -10, and ribosome binding sites (RBS). The stem-arrow structure located above the DNA sequence (base 258) indicates the start site of transcription. The inverted repeat depicted by lines with diamond ends (located below bases 261–291) indicate bases of the RAT site that are involved with the formation of a hairpin structure in the mRNA in the presence of a beta-glucoside. The inverted repeat depicted by lines with ball ends (located below bases 281–361) indicate bases that are involved with the formation of the transcription terminator in the absence of inducing beta-glucoside. Note the overlapping nature of the two inverted repeat structures. The asterisk above base 366 indicates the last residue of the short terminated transcript in the absence of inducer. The start site of translation and the amino-terminal end of the *bglP *protein are also shown below base 420.

**Figure 3:**
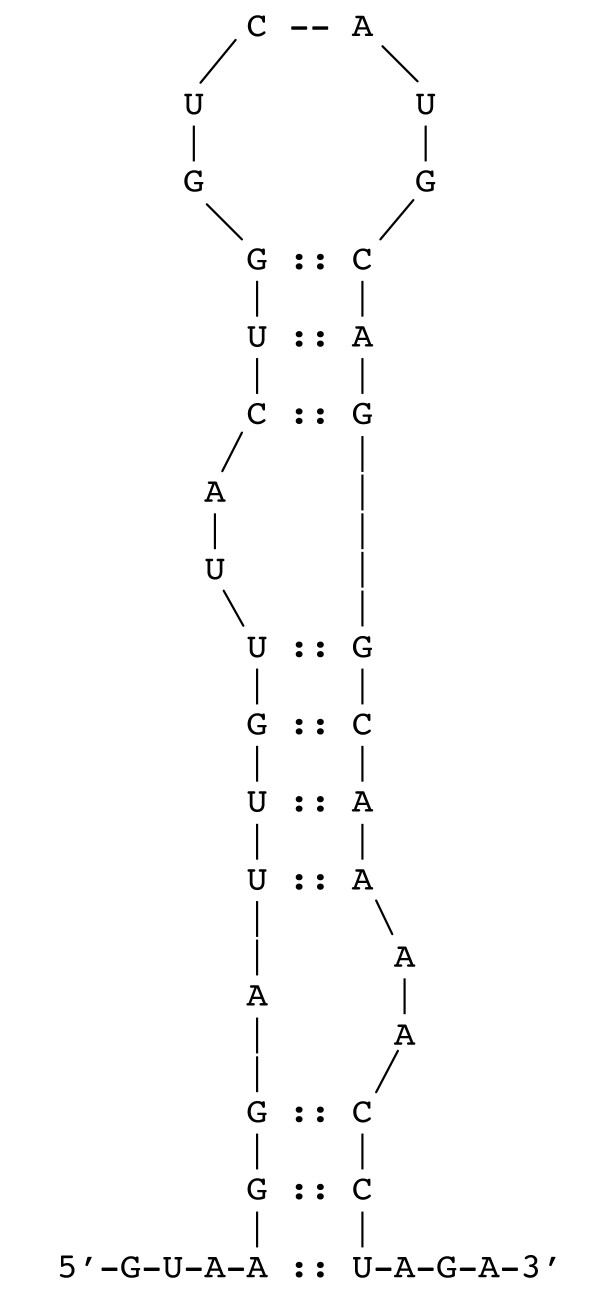
Diagram of the hairpin formed by the RAT sequence in the mRNA. The 5' end of the *bglP *mRNA is shown depicting the hairpin structure that would be bound by the LicT protein. This structure displays two bulges that are conserved in RAT hairpin structures.

The putative RAT site is highly conserved with other antitermination protein binding sites [9]. This genetic element is a region of diad symmetry that may form a hairpin structure at the 5' end of an mRNA molecule (Figure 2). This structure (Figure 3), when stabilized by the binding of a dimerized and active antitermination protein such as the LicT molecule, would inhibit transcription termination and allow transcription to proceed through the 3' gene, *bglP*.

This report provides a transcriptional analysis of the *S. mutans bglP *locus that is putatively regulated by the mechanism of transcriptional antitermination in the presence of esculin. The LicT protein is essential for efficient expression of the *bglP *gene and esculin hydrolysis by *S. mutans *in the presence of glucose. Physical examination of the *bglP *gene and its RNA transcripts has generated evidence that supports the hypothesis that the *bglP *locus is regulated by an antitermination mechanism mediated by the LicT protein. This report is the first demonstration of the antitermination process in *S. mutans*.

## Results and discussion

### Localization of the *bglP *promoter

**Figure 4:**
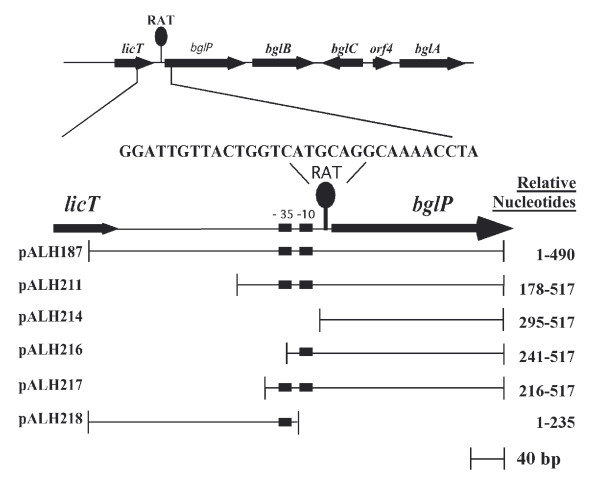
Diagram illustrating the *S. mutans *DNA fragments used in the various reporter constructs used to localize the promoter of the *bglP *gene. The position of the ribonucleic antitermination (RAT) site is also depicted. The following structures are located relative to the *Xba*I site located at the 5' end of the restriction fragment: -35 promoter region, nucleotides 224–229; -10 promoter region, nucleotides 246–251; start site of transcription, nucleotide 258; and the RAT sequence, nucleotides 262–290.

**Figure 5:**
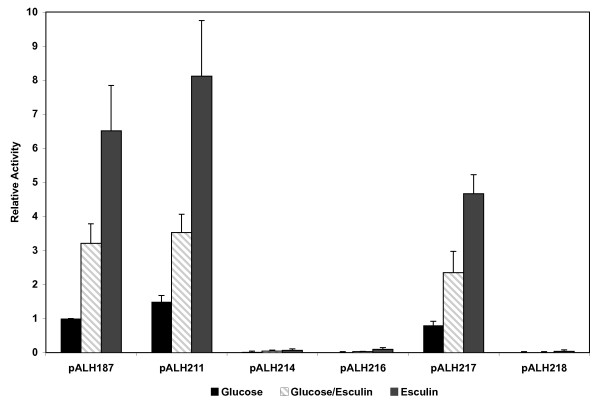
Relative promoter activity associated with various *bglP *promoter constructs. The transcriptional activity associated with the *bglP *promoter constructs diagramed in Figure 4 was determined in the wild-type *S. mutans *host NG8. Cells were grown in CDM supplemented with 0.5% of each carbohydrate prior to determination of β-galactosidase activity. The results shown are an average of at least seven experiments +/- the standard deviation.

Upon determining that the LicT protein might be involved in the regulation of *bglP *[9], experiments were undertaken to further localize the *bglP *promoter activity. Beta-galactosidase assays had previously illustrated that the 490 bp *Xba*I*-Hind*III fragment cloned in pALH187 contained regulated promoter activity. Sequence analysis of the region 5' to the *bglP *gene revealed putative consensus -35 and -10 promoter elements located within the *Xba*I-*Hind*III restriction fragment. Five additional reporter constructs were created to more precisely locate the promoter of the *bglP *gene (Figure 4). These constructs contained various deletions within the putative *bglP *promoter region and eliminated various putative promoter elements. The various deletion constructs were analyzed for their relative transcriptional activity in either the presence or absence of the inducer, esculin. As depicted in Figures 4 and 5, both the putative -35 (TTGCAA) and -10 (TATAAT) regions are required to be present in the reporter construct for any detectable transcriptional activity initiating from this region of *S. mutans *DNA. The constructs containing only one of these regions (pALH216 and pALH218) or neither region (pALH214) do not express detectable levels of β-galactosidase activity (Figure 5).

The DNA sequence from the *Xba*I-*Hind*III fragment located 5' to the region contained in pALH211 (nucleotides 1–177 relative to the *Xba*I site) did not contribute significantly to the transcriptional activity associated with the *bglP *gene. This point is observed when examining the relative activity of pALH187 and pALH211 (Figure 5). However, further deletion of the DNA toward the putative -35 region in pALH217 does affect the activity of the promoter. This construct contains only 8 bp 5' to the putative -35 region and exhibits approximately 50% of the relative activity of pALH187 and pALH211. The additional 36 bases contained in pALH211 upstream of the -35 region appear to enhance transcription from the *bglP *promoter. It was also demonstrated that the region located 3' of the -10 promoter region, as contained in pALH214, is not associated with detectable levels of β-galactosidase activity (Figure 5).

A reporter plasmid that contained only the -35 and -10 region (relative nucleotides 178–260) was also constructed. In this construct, the entire RAT sequence (relative nucleotides 262–290) was absent. This construct would be predicted to have constitutive expression because both the sequence required for transcription termination in the absence of beta-glucosides and the sequence required for antitermination in the presence of beta-glucosides were removed. This plasmid was highly unstable in *S. mutans *and underwent spontaneous deletion events (data not shown). Presumably, unfettered expression of the *E. coli *beta-galactosidase enzyme from the consensus -35 and -10 promoter regions of the *bglP *gene was toxic to the *S. mutans *cells.

**Figure 6:**
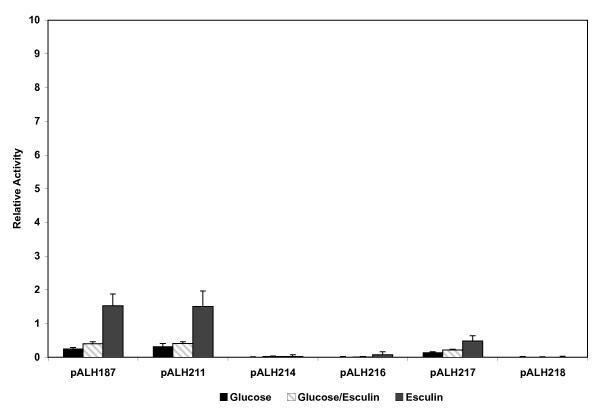
Relative promoter activity associated with various *bglP *promoter constructs in the *S. mutans *NG8 *licT*::Ω-Kan2 strain, ALH201. Growth conditions are as described for Figure 5.

In addition, this series of plasmid reporter constructs was transformed into ALH201 (*licT*::Ω-Kan2). The resultant transformants were assayed for beta-galactosidase activity. None of the plasmid constructs in the ALH201 background displayed any significant beta-galactosidase activity when compared to the activity found in the wild-type strain (Figure 6). These results confirm the in vivo fermentation data previously reported [22]. These data confirm that the LicT protein is essential for optimal transcriptional activity of the *bglP *promoter region.

### Determination of the transcriptional start site of the *bglP *gene

**Figure 7:**
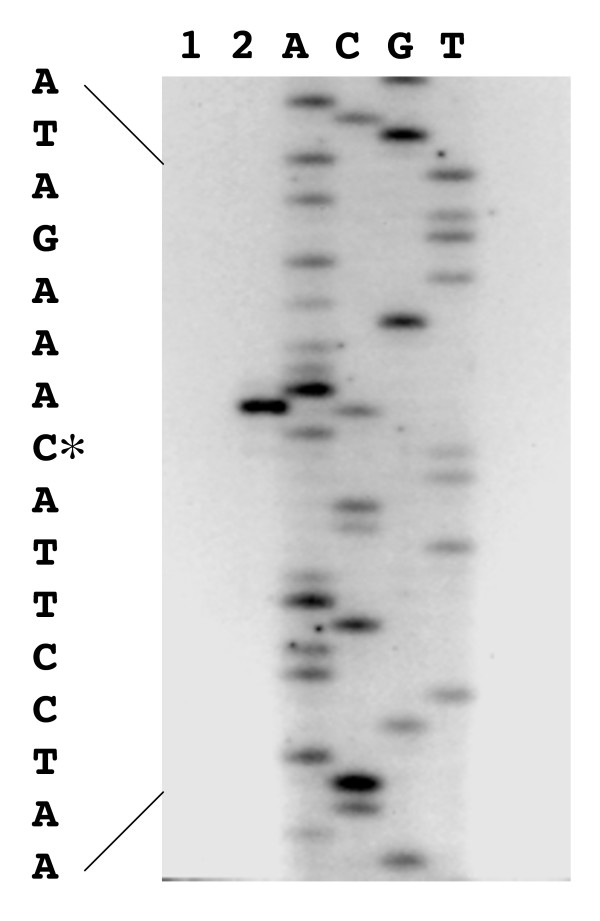
Reverse transcriptase mapping of the *bglP *transcriptional start site. The negative control (no RNA) primer extension reaction product (Lane 1) and the primer extension reaction product using *S. mutans *RNA (Lane 2) are included. The DNA sequencing reactions (A, C, G, and T) were performed on the plasmid pALH163 containing the entire *bgl *regulon.

Primer extension experiments were performed to identify the transcriptional start site of the *bglP *gene. These experiments determined that the major start of transcription mapped to a single nucleotide (Figure 7). The transcriptional start point, a single G, is 150 base pairs upstream of the putative ribosomal binding site AAGGAG and 162 base pairs upstream of the initiation codon (ATG) of the *bglP *gene. The transcriptional start point is located 7 bp 3' to the -10 promoter elements and located 4 base pairs 5' of the putative RAT sequence (Figure 2).

### Northern blot analysis of the *bglP *transcript

**Figure 8:**
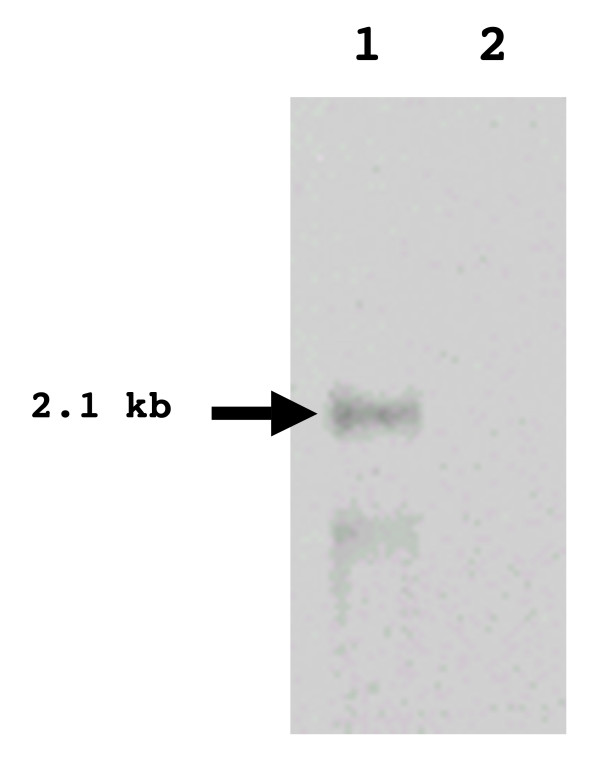
Identification of the full length *bglP *transcript. Northern blot analysis was performed on total RNA isolated from *S. mutans *(ALH213) grown in the presence of either esculin (Lane 1) or glucose (Lane 2).

To confirm previous work that suggested the *bglP *and *bglB *genes do not constitute an operon [22] and to determine the actual size of the *bglP *transcript, Northern blot analysis was performed on total RNA isolated from *S. mutans*. The Northern blot identified a transcript that corresponded with the proposed size of the *bglP *transcript, 2.1 kb (Figure 8). There were no transcripts larger than the *bglP *transcript, suggesting that the *bglP *gene and downstream *bglB *gene do not comprise an operon. These data confirm previous results obtained from β-galactosidase assays that indicated the *bglP *gene is transcribed on a monocistronic transcript [22], These data, combined with the identification of the transcriptional start site, also indicate that the *bglP *gene is not co-transcribed with the regulatory *licT *gene as it is in other beta-glucoside systems.

### Size determination of the terminated *bglP *transcript

**Figure 9:**
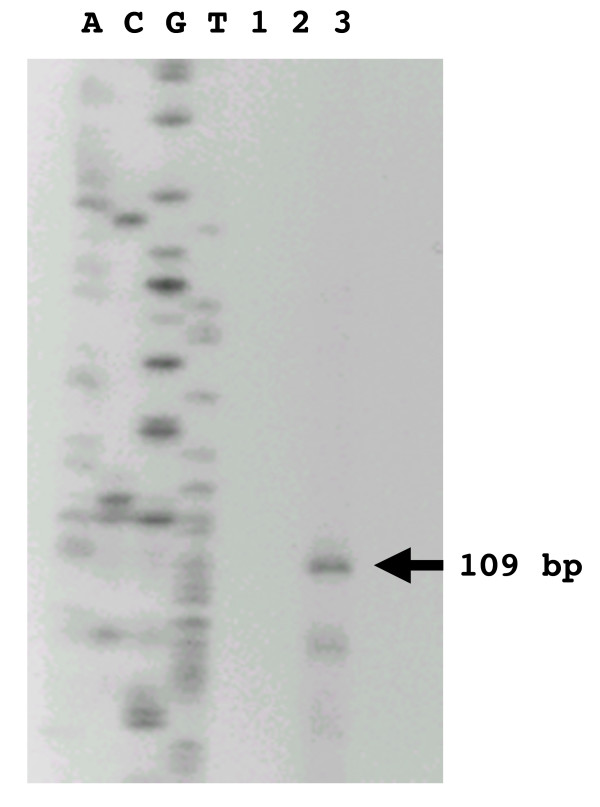
Identification of the terminated *bglP *transcript. Transcript mapping was performed on total RNA isolated from *S. mutans*. The contents of the lanes are as follows: DNA sequencing ladder in lanes A, C, G, and T; Lane 1, blank; Lane 2, negative control (no RNA); and Lane 3, transcript mapping reaction product. A 109 base transcript (indicated by the arrow) represented the only major species of RNA.

Transcript mapping experiments were undertaken to further evaluate the hypothesis that the process of antitermination regulates the *bglP *transcript and that a short, terminated *bglP *transcript would be observed in RNA collected from *S. mutans *cells grown in the absence of esculin. Mung bean nuclease transcript mapping experiments have identified a small 109 base transcript that is present in the total RNA from uninduced *S. mutans *cells (Figure 9). The identification of this terminated *bglP *transcript adds further validity to the hypothesis that antitermination regulates this locus.

## Conclusion

In this report, we have demonstrated that the *bglP *gene of *S. mutans *produces a short, truncated mRNA transcript of 109 bases in the absence of the inducer, esculin. In the presence of esculin, a 2.1 kb transcript is produced. The start site of transcription for both of these mRNA molecules is located 7 bases 3' to the -10 promoter element and 4 bases 5' to a putative RAT site at which the antiterminator protein LicT would presumably bind to allow expression of the *bglP *gene. The locations of these genetic elements are shown in Figure 1. In addition, the LicT protein has been shown to be essential for the expression of the *bglP *gene by use of transcriptional gene fusions in a *S. mutans licTi*::Ω-Kan2 genetic background. These data and the fact that the LicT protein and the RAT site are highly conserved with other putative antitermination regulatory systems imply that the LicT protein interacts with the *bglP *RAT site to control expression of this PTS permease gene.

It has been previously illustrated that the *licT *gene product regulates the expression of the *bglP *gene [9]. It was hypothesized that this regulation involved transcriptional antitermination of the *bglP *transcript. This report supports this hypothesis that the LicT antiterminator protein is regulating the *bglP *gene via binding of the RAT structure. The *S. mutans bgl *regulon has been previously shown to be unique from other beta-glucosidase operons because of several characteristics. This regulon has a novel arrangement of four adjacent genes with at least four separate transcriptional units [7]. This includes the *bglC *gene, which is located in the middle of the regulon and is transcribed on the opposite DNA strand from the other members of the regulon. The BglC protein, a member of the XylS/AraC family of transcriptional regulators, has been shown to be a positive regulator of the *bglA *gene, which encodes the phospho-beta-glucosidase [22]. This regulon has also been shown to display a glucose-resistant phenotype of catabolite repression [7]. This report now adds transcriptional regulation by antitermination to the list of interesting characteristics of this beta-glucoside-specific PTS regulon from *S. mutans*.

## Methods

### Bacterial strains and media

*S. mutans *NG8 was used as the source of chromosomal DNA for PCR reactions as well as the host strain for all *S. mutans *transformations. *E. coli *CC118 [*Δ*(*ara-leu*) 7697 *araD*139 *ΔlacX*74 *galE galK ΔphoA*20 *thi*-1 *rpsE rpoB argE*(Am) *recA*1] [23] was used for recombinant DNA procedures. *S. mutans *strains were grown on Brain-Heart Infusion (BHI) agar plates or in BHI broth (Difco, Detroit, MI). *S. mutans *strains were grown under antibiotic selection with kanamycin (500 μg/ml), erythromycin (20 μg/ml), or a combination of both antibiotics, as necessary. All *E. coli *strains were grown on Lennox Broth (LB) agar plates or in LB broth (Gibco-BRL, Rockville, MD). *E. coli *was grown under antibiotic selection with ampicillin (100 μg/ml) or chloramphenicol (20 μg/ml), as necessary. *S. mutans *cultures were grown statically at 37°C, while *E. coli *cultures were grown at 37°C with shaking aeration. Unless otherwise noted, all chemicals and reagents were purchased from Sigma (St. Louis, MO).

### DNA isolation, manipulation, and PCR amplification

Streptococcal DNA was isolated as previously described [7]. Mini-plasmid preparations were performed using the QIAprep 8 Miniprep kits or the QIAprep Spin Miniprep Kits (Qiagen, Valencia, CA). Recombinant plasmid constructs were electroporated as previously described [24] and *S. mutans *was transformed as previously described [25,26].

The *licT *gene was amplified from the chromosome of *S. mutans *NG8 by using primers designed from the sequence data obtained from the *S. mutans *genome database at the Oral Pathogen Sequence Database maintained by Los Alamos National Laboratory [27]. The primers were bglP-1 (5'-CGCAAGCGAGTAACACAGTG-3') and licT (5'-GATTAGCAAAATGAAGGCAG-3'). The annealing temperature used for this primer pair was 45°C. The KlenTaq-LA polymerase (Clontech, Palo Alto, CA) was used according to the manufacturer's recommendations. All DNA manipulations and enzyme procedures were done according to the manufacturer's instructions and under NIH recombinant DNA guidelines.

### Construction of *lacZ *fusions

The *lacZ *transcriptional fusions were created using the parent plasmid, pALH122 [28]. Briefly, this plasmid is a derivative of the shuttle vector pVA838 [29] and contains the *lacZ *gene from *E. coli *for use as a reporter of transcriptional activity. This vector can replicate in both Gram-negative and Gram-positive bacteria. It contains an erythromycin resistance marker for selection in *S. mutans *and a chloramphenicol resistance marker for selection in *E. coli*.

The construction of pALH187, which displays regulated promoter activity, has been previously reported [22], Briefly, a 490 bp *Xba*I*-Hind*III fragment, containing the entire *bglP *promoter region, was inserted into the *Sma*I site of pALH122. In order to further localize the promoter activity of the *bglP *gene, five additional reporter constructs were made. These constructs contain various segments of the putative promoter region. A diagram of the regions cloned is shown in Figure 2. In this series, various PCR fragments were cloned into the *Sma*I site of pALH122 to create different *lacZ *reporter plasmids.

All of the reporter constructs were analyzed by restriction endonuclease digestion, DNA sequencing, and/or PCR analysis. These analyses were performed to verify that a single copy of the amplified PCR fragment was cloned into pALH122 and to ensure that the desired fragment was cloned in the correct orientation to express the *lacZ *reporter gene. The reporter constructs were then transformed into *S. mutans *NG8 as described to determine their relative promoter activity in the appropriate *S. mutans *host background.

### Fluorescent beta-galactosidase assays

*S. mutans *strains containing the various *lacZ *transcriptional fusions were grown in Chemically Defined Medium (CDM) [30] supplemented with glucose, esculin, or a combination of glucose and esculin. All carbohydrates were at a final concentration of 0.5%. After being harvested by centrifugation, the cells were physically lysed with a Mini-BeadBeater (Biospec, Inc., Bartlesville, OK) and fluorescent beta-galactosidase assays were then performed as previously described [22]. Briefly, the cleared cell lysates were allowed to incubate in a reaction well containing 40 μl of AB buffer (60 mM K_2_HPO_4_, 40 mM KH_2_PO_4_, 100 mM NaCl) [31] and 10 μl of resorufin-β-D-galactopyranoside (0.4 mg/ml in DMSO) (Molecular Probes, Eugene, OR). After 30 min, the reaction was slowed by the addition of excess AB buffer. Sodium bicarbonate was added to adjust the pH of the reaction mixture to increase the sensitivity of the assay by approaching the optimal extinction coefficient of resorufin salt. Two hundred microliters of the reaction mixture was then added to a black opaque microtiter plate and read in a fluorescent plate reader (Molecular Devices, Sunnyvale, CA). The data were obtained as relative fluorescent units (RFUs). The protein concentrations of the bacterial cell lysates were determined using the Coomassie Plus Protein Assay Reagent (Pierce, Rockford, IL). The data were standardized as RFUs/μg of cell lysate protein prior to conversion to specific activities using a resorufin salt standard. The specific activity was expressed as ng resorufin salt formed/μg protein. Specific activities were divided by the activity from the *S. mutans *strain containing pALH187 grown in glucose. This served as an internal control for daily growth variation and yielded relative transcriptional activities, which were then plotted (Figure 3).

### RNA isolation

One ml of an overnight *S. mutans *NG8 starter culture was used to inoculate a 15 ml screw cap tube containing 14 ml of prewarmed CDM supplemented with various carbohydrates at 0.5% final concentration. The cultures were allowed to grow at 37°C for 6–8 hr; at this time, the cultures were approximately at the mid-log phase of growth. Cells were harvested by centrifugation and transferred to a 1.5 ml microfuge tube. The cells were washed in 1 ml of TE buffer and then resuspended in 1 ml of Trizol reagent (Gibco BRL). The microfuge tubes containing the cell/Trizol suspensions were filled approximately 1/2 full with zirconia/silica beads and physically lysed in a Mini-BeadBeater for 80 sec. The samples were placed on ice, 200 μl of chloroform added to the lysate, and the mixture agitated again for 20 sec. The samples were then microcentrifuged for 10 min at 4°C. The aqueous phase was removed and precipitated with isopropanol overnight at -20°C. The RNA pellet was washed with 100 μl of 100% ethanol and resuspended in 100 μl of DEPC-treated water. The sample was treated with RQI RNase-Free DNase (Promega, Madison, WI) as recommended by the manufacturer. The resulting RNA was purified further and concentrated using an RNeasy Total RNA kit (Qiagen).

### Primer extension reactions

Primer extension experiments were performed using the Primer Extension-AMV Reverse Transcriptase kit (Promega, Madison, WI) and *S. mutans *RNA isolated from cells grown in CDM supplemented with 0.5% esculin. A DNA primer (called Primer Extension-2) (5'-CCAAATAAATACAACAAAACACTCG-3') was end-labeled with gamma-^32^P-ATP and was used in both the primer extension and the DNA sequencing reactions using the *fmol *sequencing system (Promega). For reverse transcriptase mapping, the primer was hybridized with approximately 30 μg of RNA at 42°C and the primer extension reaction was carried out as described by the manufacturer. Di-deoxynucleotide sequencing reactions, using the same primer and a DNA template, were electrophoresed with the primer extension product on a denaturing polyacrylamide sequencing gel in order to determine the endpoints of the primer extension product.

### Size determination of the terminated *bglP *transcript

Transcript mapping was performed on *S. mutans *RNA isolated from cells grown in 0.5% lactose using mung bean nuclease (New England Biolabs, Beverly, MA). Lactose was used because it represses the *bglP *gene more than glucose does and yields more of the terminated transcript (data not shown). A radiolabeled DNA probe was generated by amplifying a 277 bp region of *S. mutans *DNA using the primers bglP-1 and LicT-3 (5'-TCTGCTATAATATCTTTGTAAGG-3'). This PCR reaction was carried out as previously described, but with the addition of 5 μl each of alpha-^32^P-dGTP and alpha-^32^P-dCTP (NEN-Perkin Elmer Life Sciences, Boston, MA). The PCR product was denatured in boiling water for 10 min and immediately chilled on ice for 10 min. This radiolabeled probe was added to 40 μg of total RNA that was isolated from *S. mutans *as previously described. The DNA probe/RNA mixture was ethanol precipitated and washed with 70% ethanol. The nucleic acid pellets were air dried for 30 min prior to resuspension in 20 μl of hybridization buffer (80% formamide, 40 mM PIPES (pH 6.4), 400 mM NaCl, and 1 mM EDTA (pH 8)) [32], The samples were denatured at 65°C for 10 min and then placed in a 40°C heat block and allowed to hybridize over night. Following hybridization, 300 μl of mung bean nuclease reaction mix (1X enzyme buffer and 500 Units/ml of mung bean nuclease) was added to each sample (New England Biolabs). The reaction tubes were incubated at 37°C for 2 hr. The mung bean nuclease was inactivated by the addition of EDTA to a final concentration of 1 mM. The mixture was ethanol precipitated and washed with 70% ethanol. The final pellet was dried with a Speed-Vac SC100 (Savant, Waltham, MA) for 5 min. The signal intensity was estimated and the products were diluted accordingly with TE buffer and formamide loading dye (Promega). The transcript mapping products were denatured in boiling water for 10 min and then chilled on ice for 10 min prior to visualization on an 8% polyacrylamide sequencing gel. The DNA ladder was produced by a sequencing reaction using the LicT-3 primer. Thus, the size of the transcript could be determined by comparing it to the ladder of the known DNA sequence.

### Northern blotting

Total RNA was subjected to Northern blot analysis. Approximately 10 μg of total *S. mutans *RNA (final volume of 11 μl) was combined with 9 μl of 12.3 M formaldehyde, 5 μl of 10X MOPS running buffer (0.4 M MOPS (pH 7.0), 0.1 M sodium acetate, and 0.01 M EDTA), and 25 μl formamide. The samples were then incubated at 55°C for 15 min before being loaded onto a denaturing formaldehyde gel. The formaldehyde gel was composed of 1% of agarose, IX MOPS running buffer, and 6.6% formaldehyde. The total RNA was run on the denaturing formaldehyde gel at approximately 100 volts until the bromphenol blue dye had migrated approximately 2/3 the length of the gel. The gel was then blotted onto a positively charged nylon membrane (PALL Corporation, Ann Arbor, MI) using the Genie Electrophoretic Blotter (Idea Scientific, Minneapolis, MN). As suggested by the manufacturer, the blotting was performed at 12 volts for two hrs in Northern transfer buffer (20 mM MOPS, 5 mM sodium acetate, and 0.5 mM EDTA). The membrane was then washed with 0.2X SSC for 5 min, gently dried, and the RNA crosslinked to the membrane using a Stratalinker UV generator (120,000 microjoules) (Stratagene, La Jolla, CA).

The membrane was stained with 0.03% (w/v) methylene blue in 0.3 M sodium acetate for 45 sec. The membrane was then destained with de-ionized H_2_O. Membrane staining was performed to identify the ribosomal RNA bands. The image of the stained membrane was electronically scanned for future reference. The membrane was then permitted to prehybridize for at least 1 hr at 55°C in a Hybaid Hybridization oven (Labnet Systems, Edison, NJ). The North2South Northern Blot kit (Pierce) was used as described by the manufacturer to identify the transcripts of interest. The DNA probe was generated using the same PCR primers that were used for probe generation in the transcript mapping experiments. However, the probe was generated using biotinylated nucleotides instead of being radiolabeled. This allowed for non-radioactive, chemiluminescent detection of the transcripts on the Northern blot. The biotinylated probe was detected with the Neutravidan antibody and developed with luminol enhancer and stable peroxide solution as described in the methodology for Southern blots (Pierce).

## Authors' contributions

CKC carried out the experiments and drafted the original manuscript. ALH conceived the study and crafted the final manuscript. All authors read and approved the final manuscript.
